# Targeting the antigen processing and presentation pathway to overcome resistance to immune checkpoint therapy

**DOI:** 10.3389/fimmu.2022.948297

**Published:** 2022-07-22

**Authors:** Silvia D’Amico, Patrizia Tempora, Ombretta Melaiu, Valeria Lucarini, Loredana Cifaldi, Franco Locatelli, Doriana Fruci

**Affiliations:** ^1^ Department of Paediatric Haematology/Oncology and of Cell and Gene Therapy, Bambino Gesù Children’s Hospital, Istituto di Ricovero e Cura a Carattere Scientifico (IRCCS), Rome, Italy; ^2^ Department of Clinical Sciences and Translational Medicine, University of Rome “Tor Vergata”, Rome, Italy; ^3^ Academic Department of Pediatrics (DPUO), Bambino Gesù Children Hospital, Istituto di Ricovero e Cura a Carattere Scientifico (IRCCS), Rome, Italy; ^4^ Catholic University of the Sacred Heart, Rome, Italy

**Keywords:** immunopeptidome, tumor antigens, antigen processing, HLA class I, immune checkpoint inhibitors, TEIPPs, ERAP1/2, cancer immunotherapy

## Abstract

Despite the significant clinical advances with the use of immune checkpoint inhibitors (ICIs) in a wide range of cancer patients, response rates to the therapy are variable and do not always result in long-term tumor regression. The development of ICI-resistant disease is one of the pressing issue in clinical oncology, and the identification of new targets and combination therapies is a crucial point to improve response rates and duration. Antigen processing and presentation (APP) pathway is a key element for an efficient response to ICI therapy. Indeed, malignancies that do not express tumor antigens are typically poor infiltrated by T cells and unresponsive to ICIs. Therefore, improving tumor immunogenicity potentially increases the success rate of ICI therapy. In this review, we provide an overview of the key elements of the APP machinery that can be exploited to enhance tumor immunogenicity and increase the efficacy of ICI-based immunotherapy.

## Introduction

The impressive clinical results achieved with immunotherapy in terms of remission and improved survival have given new optimism for treating patients with cancer (1, 2). However, the number of patients in whom the benefict of treatment are lasting and resolving is limited.

Identifying the mechanisms adopted by the tumor to escape immunologic surveillance becomes of paramount importance to improve the efficacy of immunotherapy. The cancer immune cycle describes the sequence of events by which an antitumor immune response results in the effective killing of cancer cells (3). In the first step, tumor antigens present in the tumor microenvironment are captured by antigen-presenting cells (APC), such as dendritic cells (DCs), which migrate to draining lymph nodes to present antigens to naïve T lymphocytes *via* MHC class I and MHC class II molecules. This results in the activation of immature T cells into effector T cells which subsequently reach the tumor site through the bloodstream to specifically recognize and eliminate tumor cells (3). Because each step of the cycle contributes to tumor cell killing, inhibition of one or more of these processes may cause attenuation of antitumor responses or immune escape (4). In this regard, failure of ICI therapy could also result from a defect in any of the above-mentioned steps, i.e., insufficient generation of antitumor T cells, inadequate function of tumor-specific T cells (5, 6), or impaired T-cell memory formation (7, 8). Recently, it has been shown that impaired migration of DCs from tumor tissues to the regional lymph nodes caused defective antigen presentation and priming of T cells, leading to uncontrolled tumor development and resistance to ICIs in several cancer types (9–12). Mechanisms related to immune escape in the cancer immune cycle as well as therapies to overcome immune escape and resistance to ICI therapies have been widely addressed (8, 13, 14).

**Figure 1:**
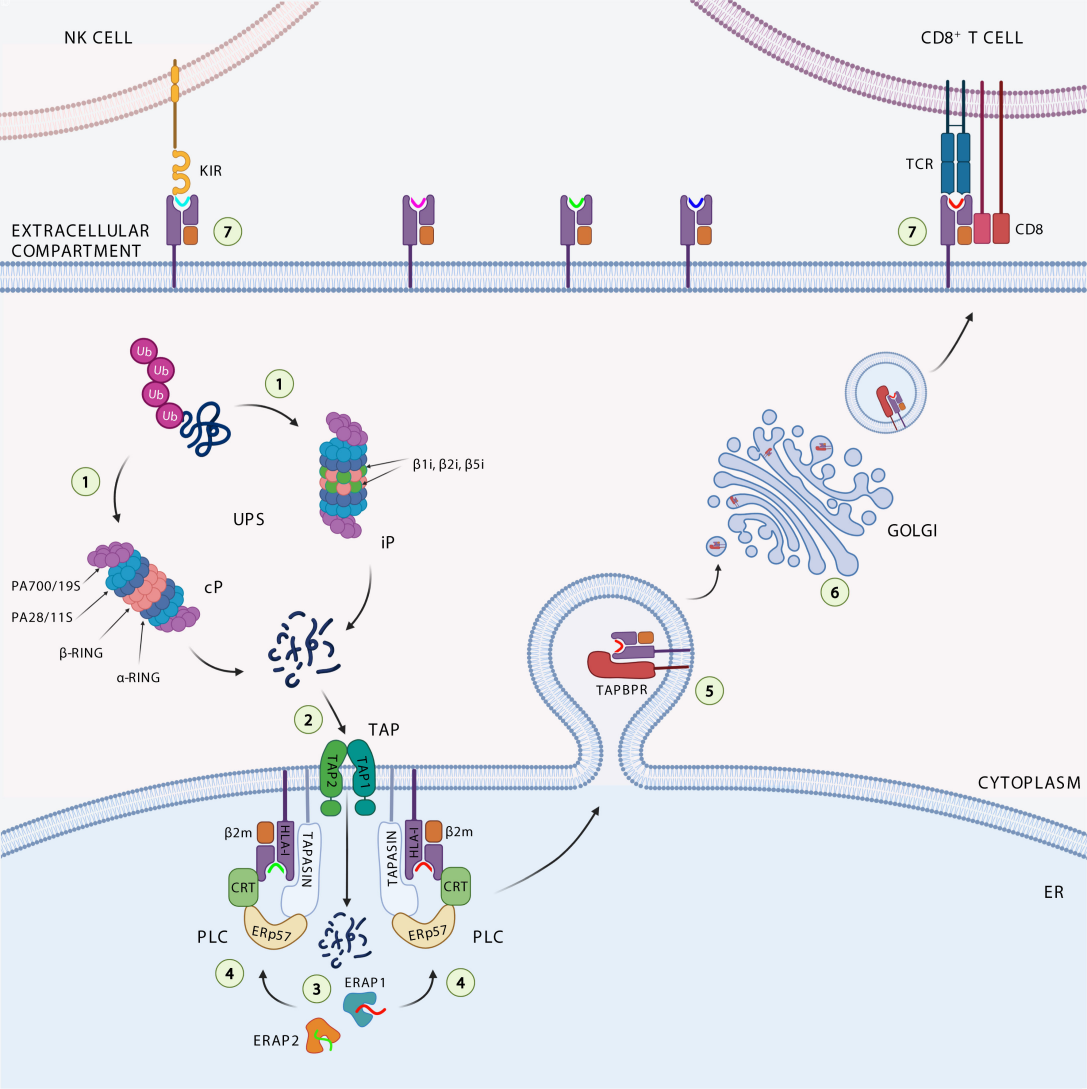
Antigen processing and presentation pathway. The ubiquitinated proteins are degraded (1) by the catalytic subunits of the constitutive proteasome (cP) or immunoproteasome (iP) into peptides. The peptides generated are translocated (2) through the transporter associated with antigen processing (TAP1 and TAP2) complex into the endoplasmic reticulum (ER), where they can be further trimmed (3) by the ER aminopeptidases ERAP1 and ERAP2. Loading onto the HLA class I heavy chain-associated β2-microglobulin (β2m) (4) is a multistep process facilitated by the peptide loading complex (PLC), composed of TAP, ERp57, and the chaperones tapasin and calreticulin (CRT). Binding of high-affinity peptide induces dissociation of the HLA class I complex from PLC, its stabilization by interaction with TAP-binding protein related (TAPBPR) (5), and subsequent trafficking (6) to the cell surface to be presented to cytotoxic CD8^+^ T cells and NK cells (7).

ICI-based immunotherapy acts by blocking the inhibitory action of immune checkpoints and restoring the function of tumor-infiltrating immune cells (15). CD8^+^ T cells are one of the main immune cell populations targeted by ICI-based therapy. These cells continuously scrutinize the integrity of the proteome by scanning peptides presented on the cell surface bound to HLA class I molecules (**Figure 1**). The integrity of this process, called antigen processing and presentation (APP) pathway, is critical to ensure generation of effective CD8^+^ T cells to fight cancer (16). The APP pathway includes several closely related proteins, whose level of expression has been shown to influence quantitatively and qualitatively the repertoire of tumor antigens presented to the immune system. Changes in the expression of individual components of the APP machinery have been associated with the lack or reduced presentation of tumor antigens, as well as variation in the level of tumor-infiltrating CD8^+^ T cells and response to ICI-based therapy (17–19). Recently, Maggs and colleagues (20) provided a detailed overview of the structural and nonstructural mechanisms underlying HLA class I APP defects in malignant cells. In this review, we deepen the contribution of APP machinery to the cancer immunopeptidome, with a focus on how they can be exploited to enhance tumor immunogenicity.

## Antigen processing and presentation machinery

Tumor antigens are small pieces of protein able to provoke an immune attack against cancer. They can be derived from mutated or unmutated cellular proteins and are referred to as tumor-specific antigens or tumor-associated antigens, respectively (21). Several lines of evidence indicate that potential sources of tumor antigens could also arise from the translation of presumably non-coding transcripts, such as introns, untranslated regions, intergenic sequences, or out-of-frame exons (22–25). In general, peptides presented by HLA class I molecules are not necessarily derived from the most highly expressed proteins, such as housekeeping proteins (histones, ribosomal proteins, metabolic enzymes, and cytoskeletal proteins) (22, 23, 26). Indeed, unstable proteins, i.e., those that are rapidly degraded by the proteosome, are also represented in the immunopeptidome. Such proteins, although hardly detectable by mass spectrometry analysis, are in fact able to generate immunogenic peptides (22, 23, 26). Regardless of their origin, peptides compete with each other for presentation by HLA class I molecules (22, 26, 27). It has been estimated that the immunopeptidome, composed of thousands of short peptide antigens of 8-12 amino acids, contains at most 1% of the peptide sequences found in individual cells.

The intracellular antigen processing pathway almost exclusively deals with the ubiquitinin proteasome system (UPS) activity, by which endogenous proteins are first ubiquitinated and subsequently degraded by the proteasome, a large cytosolic proteolytic complex which digests unneeded or damaged proteins (**Figure 1**) (28). Proteasome complex consists of 14 structural subunits and 2 copies of 3 catalytic subunits β1, β2, and β5 (29). Following stimulation with interferon-γ (IFNγ), due to cellular stress and inflammation, the constitutive proteasome is converted in the immunoproteasome, which differs in the catalytic subunits β1i (LMP2 or Psmb9), β2i (MECL1 or Psmb10) and β5i (LMP7 or Psmb8) (30). The immunoproteasome shows increased peptide cleavage activity after basic or hydrofobic residues, allowing the production of peptides with higher affinity for HLA class I molecules (31–33). A fraction of these peptides are transported into the lumen of the endoplasmic reticulum (ER) by the transporter associated with antigen processing (TAP), a heterodimeric complex belonging to the ABC transporter superfamily. TAP is composed of two subunits (TAP1/TAP2), both consisting of a transmembrane region, a substrate binding site, and an ATP-binding domain, and preferentially interacts with peptides (one at a time) ranging from 8 to 16 amino acids in length (34). These properties give TAP the ability to perform an initial selection of peptides available for HLA class I binding. It has been estimated that about 25% of antigenic peptides reaching the cell surface bound to HLA class I molecules are shortened at the N-terminal end by ER aminopetidases ERAP1 and ERAP2 (ERAAP in mouse), which are known to generate and destroy peptides (35–37). Peptides with the correct length and sequence to bind HLA class I alleles are loaded onto β2m-associated HLA class I heavy chain dimers with the help of the peptide-loading complex (PLC) that consists of TAP, ER resident protein 57 (ERp57), and chaperone molecules calreticulin and tapasin. The peptide-HLA class I-β2m complex formed is further stabilized by the binding with the tapasin-binding protein related (TAPBPR), the newest member of the APP machinery (**Figure 1**) (38). Tapasin and TAPBPR work together sequentially, with tapasin involved in loading the peptide into the HLA class I-β2m dimer within the PLC in the ER, and TAPBPR assigned to control the stability of the peptide-HLA class I-β2m complex in the Golgi during its transport across the plasma membrane (39). The resulting trimeric complex travels to the plasma membrane to be presented to cytotoxic CD8^+^ T cells and NK cells.

Defective expression of APP components has detected in most of the tumors. Many evidence indicates that impaired functionality of the APP machinery in tumor cells, in addition to causing a change in the repertoire of antigens presented on the cell surface, results in a change in the level of tumor-infiltrating CD8^+^ T cells and the response to ICI-based therapy.

## Contribution of the antigen processing and presentation machinery to the cancer immunopeptidome

### Immunoproteasome

Several studies have shown that mice lacking the single catalytic subunits of the immunoproteasome have relatively modest changes in the immunopeptidome (40–42). However, mice completely lacking immunoproteasome had defects in the presentation of several MHC class I epitopes with an immunopeptidome 50% difference from wild type mice (43). These differences were sufficient to stimulate robust rejection of wild type splenocytes transplanted in triple knock-out mice (43), thus suggesting that the immunoproteasome plays a primary role in antigen presentation. Based on its ability to modulate the expression of pro-tumorigenic cytokines and chemokines or increase the presentation of tumor antigens, the immunoproteasome shows both pro- and anti-tumor properties, respectively (44). The pro-tumoral role of the immunoproteasome is evident in the case of colitis-associated carcinogenesis, where increased expression of β5i/LMP7 and β1i/LMP2 has been observed in cronically inflamed colons (45). As a result, β5i/LMP7-deficient mice show reduced tumor formation and production of pro-tumor chemokines (e.g. CXCL1, CXCL2, and CXCL3) (45). In contrast to the inflammatory environment of the gut, increased expression of β5i/LMP7 and β1i/LMP2 subunits in melanoma cells resulted in an altered immunopeptidome which correlated with increased reactivity of tumor-infiltrating CD8^+^ T cells, improved survival and response to ICIs (46). Indeed, increased immunoproteasome expression has been attributed to IFNγ secretion by tumor-infiltrating CD8^+^ T cells in several tumor types, including melanoma, colorectal cancer, gastric cancer, and breast cancer (46–50). Similarly, in non-small cell lung cancer (NSCLC), high immunoproteasome expression was correlated with good prognosis (51). In this scenario, induction of immunoproteasome expression could be an interesting approach to improve the effect of ICI-based therapy in specific tumor settings.

### TAP

Several viruses and tumors exploit the down-regulation or inhibition of TAP as a strategy to evade CD8^+^ T-cell control (52). In this context, it has been observed that TAP-deficient cells display a severe reduction of HLA class I surface expression, but still sufficient to have a partial antigen presentation and functional T-cell recognition through alternative processing pathways (53). In TAP-deficient tumor cells, although the presentation of conventional tumor-specific antigens may decrease, an alternative class of non-mutated antigens emerges within the immunopeptidome. These novel antigens, called T cell epitopes associated with impaired peptide processing (TEIPPs), derive from normal housekeeping proteins and are not loaded on HLA class I molecules in healthy cells. Accordingly, in mouse and human models, CD8^+^ T cells of healthy individuals specific for some TEIPP antigens reside in the naïve state, indicating that they are not triggered during an immune response (53, 54). Conversely, as demonstrated by a number of studies, TEIPPs are efficient in elicit a functional antitumor CD8^+^ T cell response (53, 55, 56). Marijt and colleagues identified a human HLA-A2-binding TEIPP derived from the LDL Receptor Related Protein Associated Protein 1 (LRPAP1) signal sequence (LRPAP1_21-30_) common to different type of tumors, including melanoma, lymphoma, colon and renal cell carcinoma (53). The authors demonstrated that LRPAP1_21-30_-specific CD8^+^ T cells selectively recognized TAP-defective cancers, but not the TAP-expressing counterparts on healthy tissue (53). More recently, the same research group designed a synthetic long peptide vaccine by optimizing a peptide sequence based on the previously identified LRPAP1-derived TEIPP (57). The resulting vaccine was able to induce CD8^+^ T-cell immunity and cross-presentation by monocyte-derived DC, resulting in tumor control (57). Interestingly, Garrido and colleagues used a clinical applicable method to increase the antigenicity of tumor cells by downregulating TAP expression *in situ*. The authors demonstrated that administration of TAP siRNA conjugated to a broad-spectrum tumor-targeting nucleolin aptamer resulted in: i) inhibition of tumor growth in multiple transplanted, orthotopic and autochthonous mouse tumor models of different origin and genetic background without measurable toxicity, ii) enhancement of the antitumor effect of PD-1 antibody, and iii) induction of TAP-independent peptide presentation in human tumor cells (55). Thus, treatment with the siRNA-conjugated nucleolin-TAP aptamer represents a widely applicable approach to increase the antigenicity of tumor lesions and improve the efficacy of ICI-based therapies. These studies offer different strategies to exploit TAP inhibition as an approach to make tumor cells responsive to ICI-based therapy. If in the first case, a TAP-deficient tumor proves to be more sensitive to a therapy based on TEIPP targeting, in the second one the alteration of antigen processing is used as a novel therapeutic approach to target tumor cells.

### TAPBPR

Silencing of TAPBPR in HeLa cells causes an increase in the overall number of peptides presented on the cell surface by HLA class I molecules compared with control cells (39). This suggests that inhibition of TAPBPR, by reducing affinity requirements for peptide binding to HLA class I molecules, could expand the repertoire of antigens presented on the cell surface, thereby eliciting an immune response. Recently, Ilca and colleagues demonstrated that the plasma membrane-targeted or recombinant soluble form of TAPBPR retain their peptide-editing capacities (58). Addition of recombinant TAPBPR to the extracellular environment promotes the exchange of peptides bound to HLA class I molecules directly at the plasma membrane. The authors suggest that a similar approach could be useful to enrich tumor cells with highly immunogenic peptides, inducing immune recognition of tumors and thus potentially improving immunotherapy (58). It is now clear that, as with other components of the APP, the binding affinity of TAPBPR varies among HLA class I allotypes with a strong preference for HLA-A (59). This implies that patients with different HLA class I typing might be susceptible to TAPBPR chaperone-mediated peptide editing to different degrees. This could play a role in disease susceptibility, but also influence patients’ response to possible TAPBPR-mediated therapy (60).

### ERAP1 and ERAP2

The first evidence that loss of ERAP1 function causes a profound alteration of the immunopeptidome able of eliciting potent immune responses comes from pioneering studies in mouse models of Shastri and other groups (61–64). The authors demonstrated that wild-type mice respond vigorously to the injected ERAAP^-/-^ splenocytes by activating the response of CD8^+^ T cells that specifically recognize peptides normally destroyed by ERAAP (61). Given the key role of ER aminopeptidases in the generation of the cancer immunopeptidome, several studies have explored the possibility of targeting ER aminopeptidases for generating protective anticancer responses. The first evidence comes from Cifaldi and colleagues, who demostrate that ERAAP silencing leads to rejection of murine RMA T-cell lymphoma in syngeneic mice (65). This tumor rejection was due to both T cells and NK cells, and was dependent on the repertoire of peptides bound to MHC class I molecules. Indeed, replacement of endogenous peptides with high-affinity peptides was sufficient to restore a protective effect through recognition of the stable peptide-MHC class I complex by NK cell inhibitory receptors (65). Similarly, inhibition of human ERAP1 was also able to regulate NK cell activity by controlling the interaction of peptide-HLA class I complexes with NK cell inhibitory receptors (66, 67). James and colleagues assessed the role of ERAAP expression in a syngeneic model of colorectal cancer and demonstrated that the immunodominant peptide GSW11 is trimmed and destroyed by ERAAP (**Table 1**). As expected, inhibition of ERAAP by either gene silencing or drug treatment, caused an increase of GSW11 presentation and reduced tumor growth in syngeneic mice (68).

**Table 1:** Tumor antigens affected by ERAAP/ERAP1.

Peptide	Sequence	MHC class I	Origin	ERAP1 effect	Tumor	Ref.
GSW11	GGPESFYCASW	H-2D^d^	gp90	destroyed	CRC*	(56)
MART-1_26-35_	EAAGIGILTV	HLA-A*02:01	MART-1	destroyed	MEL	(57)
HPV E7_82-90_	LLMGTLGIV	HLA-A*02:01	HPV E7	generated	OPSCC	(61)
gp100_209-217_	ITDQVPFSV	HLA-A*02:01	gp100	generated	MEL	(58)

*CRC, colorectal carcinoma; MEL, melanoma; OPSCC, oropharyngeal squamous cell carcinomas

The involvement of ERAP1 in the generation and destruction of tumor antigens has been endorsed by many other studies. Keller and colleagues demonstrated that constitutive expression of ERAP1 and proteasome activator 28 (PA28) were sufficient to inhibit generation of the MART-1_26-35_ epitope (69) (**Table 1**). The authors show that both genetic and pharmacological inhibition of ERAP1 strongly increase MART-1_26-35_ presentation in human melanoma cells and IFN-γ release by MART-1_26-35_-specific CD8^+^ T cells (69). Textoris-Taube and colleagues demonstrate that ERAP1, but not ERAP2, is involved in the generation of the glycoprotein 100 (gp100)_209-217_ immunogenic epitope derived from melanoma differentiation antigen (gp100)^PMEL17^, and promotes the activation of gp100_209-217_-specific CD8^+^ T cells by melanoma cells (**Table 1**) (70). Administration of the gp100 peptide combined with ipilimumab, the CTLA-4 antagonist, showed no improvement in disease progression in a proportion of patients with metastatic melanoma compared with those treated with ipilimumab alone, most likely due to insufficient epitope presentation (71, 72). It is possible that the allelic status of *ERAP1* known to affect its enzymatic activity and ability to generate and/or destroy antigenic peptides, may contribute to the variability of immune responses between individuals. Indeed, Reeves and colleagues demonstrated that the functional activity of different *ERAP1* allotypes is positively correlated with the amount of tumour-infiltrating CD8^+^ T cells in HPV^+^ oropharyngeal squamous cell carcinomas (OPSCC) through the generation of HPV E6/E7 epitopes (73). This is the first evidence that different *ERAP1* allotypes affect the HPV-16 epitope presentation and anti-HPV T-cell responses (**Table 1**) (73).

These studies collectively suggest that inhibition of ERAP1 activity, by resulting in a novel immunopeptidome, could represent a viable therapeutic strategy to enhance protective anti-tumor immune responses. Consistently, Koumantou and colleagues demonstrated that pharmacological inhibition of ERAP1 in a melanoma cell line induced profound changes in both the quality and quantity of one half of the peptides presented, specifically increasing the presentation of peptides with high binding affinity for HLA class I molecules (74). Moreover, inhibition of ERAAP by nucleolin-targeted siRNA was able to elicit an efficient antitumor response by sensitizing transplantable 4T1 breast carcer cell model to anti-PD-1 immunotherapy (55).

Proteomic studies revealed that ERAP2, when expressed, also contributes to the immunopeptidome (75). In the tumor contest, Temponeras and colleagues showed that pharmacological inhibition of ERAP2 alters the immunopeptidome in the MOLT-4 human leukemia cell line, with more than 20% of peptides detected as novel, or significantly up-regulated (76). Most of these peptides were 9mers with sequence motifs consistent with optimal binding motifs for at least one of the HLA class I alleles carried by MOLT-4 (76). Such peptides might be able to induce novel cytotoxic CD8^+^ T-cell responses against tumor cells and synergize with ICI-based therapy.

## Discussion

The immunopeptidome is the representation on the cell surface of what is actively translated and degraded within the cell, i.e., the element through which immune cells are able to detect and eliminate cancer cells (77–79). Indeed, loss of expression of HLA class I and APP components is one of the best-known mechanisms exploited by tumors to evade immune surveillance. The resulting reduced tumor antigen presentation has been associated with resistance to ICI therapy (20). However, there is a small percentage of patients in which ICI can still work despite of APP alterations, suggesting that, especially for tumors with high mutation burden, antigen presentation is not completely abolished and can represent an Achilles’ heel of the tumour (80).

**Figure 2:**
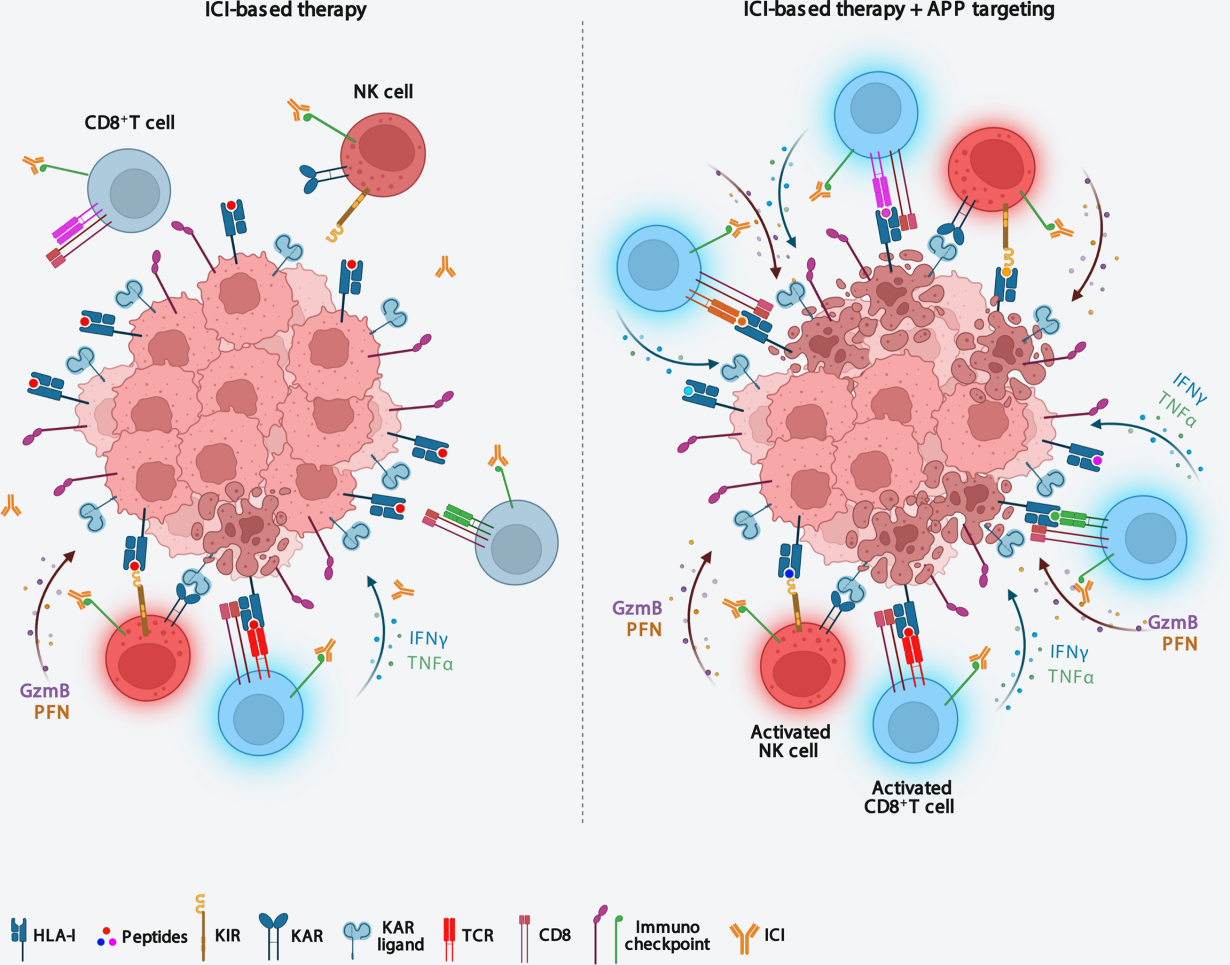
Targeting antigen processing and presentation machinery immunosensitizes ICI-resistent tumors. Downmodulation of APP components causes both an increase in tumor immunogenicity and the recall of functional effector CD8^+^ T cells and NK cells into the tumor microenvironment, thus making tumors sensitive to ICI-based immunotherapy. APP, antigen processing and presentation; ICI, Immune checkpoint inhibitor; KIR, killer inhibitory receptor; KAR, killer activating receptor.

Nevertheless, alterations in components of the APP machinery in tumors can potentially change the repertoire of peptides presented by HLA class I complexes. Accordingly, preclinical studies suggest that cells with an altered immunopeptidome due to ERAP1 or TAP deficiency still elicit strong T cell responses (55, 61). These observations indicate that a similar alteration of the immunopeptidome, by promoting the presentation of novel tumor antigens, could increase tumor immunogenicity making anticancer therapeutic protocols more effective (**Figure 2**). In this regard, there is much evidence in favor of the hypothesis that functional alteration of any component of the APP that can result in a novel immunopeptidome, could increase reactivity of tumor-infiltrating CD8^+^ T cells and sensitize tumors to ICI therapy (**Figure 2**). Interestingly, two recent *in vivo* CRISPR-Cas9 genome editing studies have demonstrated that deletion of APP-related genes is able to increase the efficacy of ICI-based immunotherapy in melanoma and renal carcinoma (81, 82). The influence of APP components on cancer cell sensitivity to immune pressure differs between the two cancer models, with ERAP1, calreticulin and TAPBPR more relevant in melanoma, and β2m in renal carcinoma (81, 82). The ability of APP component depletions to sensitize two tumor models to ICI immunotherapy (81, 82), strengthens the idea that their pharmacological inhibition may have therapeutic value in these tumors. In melanoma, it has been shown that about 12% of tumor-infiltrating T cells recognise non-mutated tumour antigens, i.e. peptides encoded by canonical exons (83).

Perturbation of APP in tumors may represent a strategy to elicit the presentation of both non-mutated tumor antigens with strong antitumor potential, as demonstrated by TEIPPs, and immunogenic epitopes usually destroyed by ERAP1 (53, 55, 57, 68, 69). This subset of peptides, considered as ‘altered self’, represents a very interesting category, as it is potentially shared by several tumors genetically or pharmacologically inhibited for APP components (55, 84).

Overall, it is clear that APP perturbation, by increasing tumor immunogenicity and widening the range of tumor antigens presented could provide an excellent opportunity to stimulate the immune response and increase the efficacy of to ICI therapy. Thus, future investigations and additional pre-clinical studies in this area are needed to reveal new and exciting anticancer therapeutic opportunities.

## Funding

This work was supported by grants awarded by Associazione Italiana Ricerca sul Cancro (AIRC) IG18495 and IG24345, H2020-MSCA-ITN-2020 Capstone-954992, Ministero della Salute Ricerca. Ricerca Finalizzata PE-2011-02351866, and Ricerca Corrente (DF).

## Conflict of interest

The authors declare that the research was conducted in the absence of any commercial or financial relationships that could be construed as a potential conflict of interest.

## Publisher’s note

All claims expressed in this article are solely those of the authors and do not necessarily represent those of their affiliated organizations, or those of the publisher, the editors and the reviewers. Any product that may be evaluated in this article, or claim that may be made by its manufacturer, is not guaranteed or endorsed by the publisher.
